# Automatic Modulation Classification of Digital Communication Signals Using SVM Based on Hybrid Features, Cyclostationary, and Information Entropy

**DOI:** 10.3390/e21080745

**Published:** 2019-07-30

**Authors:** Yangjie Wei, Shiliang Fang, Xiaoyan Wang

**Affiliations:** Key Laboratory of Underwater Acoustic Signal Processing of Ministry of Education, Southeast University, Nanjing 210096, China

**Keywords:** modulation classification, digital communication signals, cyclostationary, information entropy, SVM

## Abstract

Since digital communication signals are widely used in radio and underwater acoustic systems, the modulation classification of these signals has become increasingly significant in various military and civilian applications. However, due to the adverse channel transmission characteristics and low signal to noise ratio (SNR), the modulation classification of communication signals is extremely challenging. In this paper, a novel method for automatic modulation classification of digital communication signals using a support vector machine (SVM) based on hybrid features, cyclostationary, and information entropy is proposed. In this proposed method, by combining the theory of the cyclostationary and entropy, based on the existing signal features, we propose three other new features to assist the classification of digital communication signals, which are the maximum value of the normalized cyclic spectrum when the cyclic frequency is not zero, the Shannon entropy of the cyclic spectrum, and Renyi entropy of the cyclic spectrum respectively. Because these new features do not require any prior information and have a strong anti-noise ability, they are very suitable for the identification of communication signals. Finally, a one against one SVM is designed as a classifier. Simulation results show that the proposed method outperforms the existing methods in terms of classification performance and noise tolerance.

## 1. Introduction

Automatic modulation classification (AMC) of digital communication signals has now become an established research area [1]. It plays an important role in many applications. Some of these applications are for civilian purposes such as signal confirmation and spectrum management. The others are for military purposes such as surveillance, electronic warfare, and threat analysis. Therefore, if the types of the enemy signals are recognized, it will be of great significance for us to analyze and interfere with the enemy information.

Many methods for the modulation recognition of the communication signals have been published in recent years. In general, these methods can be divided into two categories: one is based on the decision-theoretic framework and the other is based on the statistical pattern recognition. The decision-theoretic approach is made by maximizing the probability of a certain modulation being sent given the received signal. The maximum likelihood algorithm is the most popular algorithm used in this approach. In the pattern recognition approach, the decision is made based on a set of features extracted from the intercepted signal, which is widely used in practical engineering. Extracting features from the intercepted signal is often followed by a pattern recognizer that determines the signal modulation. The following is an overview of some of these modulation recognition algorithms.

In [2], Kim et al. develop a modulation recognition method of MPSK signals based on the decision-theoretic approach. This method is less robust and lots of prior information such as carrier frequency, initial phase, and symbol rate are all assumed to be available to the classifier. Nandi et al. [3] follow the statistical pattern recognition approach and use the instantaneous features to discriminate the communication signals. These communication signals include 2ASK, 4ASK, 2PSK, QPSK, 2FSK, 4FSK. The classifier is a tree classifier and through simulations, they demonstrate that this recognizer performed well when the SNR is greater than 15 dB. The success recognition rate is >88% for these signals at the SNR of 15 dB. This approach is easy to implement and does not need any prior information, thus making it widely used. However, the features extracted by this method is sensitive to the noise and interference, and the method also needs to set a fixed decision threshold, which is often selected empirically. The shortcomings mentioned above have seriously affected the recognition performance of this method. In [4], three layered deep neural networks have been employed for the classification of BPSK, QPSK, 8PSK, 16QAM, and 64QAM with 21statistical features. The method can achieve >90% classification accuracy when the SNR is greater than 10 dB. In [5], Afan Ali et al. develop a method for automatic modulation classification using the deep learning architecture in a combination of the In-phase and Quadrature constellation points of the received signal as the training example. The recognition rate of BPSK, 4QAM, 16QAM, 64QAM by this method is >90% when the SNR is greater than 5 dB. In [6], Weihua Jiang et al provide a modulation recognition method of non-cooperation underwater acoustic communication signals using principal component analysis and an artificial neural network. The recognition rate of the BPSK, QPSK, MFSK by this method is >91% when the SNR is greater than 5 dB. Although this method has good performance, it cannot distinguish between 2FSK and 4FSK. In [7], the modulation of the communication signals is recognized by the wavelet transform. The percentage of correct identification for PSK signals is >80% when SNR> 6 dB, and the percentage of correct identification for FSK signals is >80% when SNR> 12 dB. Although this method is a good tool to identify PSK and FSK, how to choose the appropriate wavelet function is a difficult problem to solve. Although many methods of modulation recognition have been proposed in the past [1,2,3,4,5,6,7,8,9,10,11,12,13,14,15], as far as we know, the methods to identify BPSK, QPSK, 2FSK, 4FSK and MSK signals have been rarely proposed so far. Therefore, this paper will focus on the identification of these signals.

In recent years, information entropy has been widely used in signal recognition [16,17]. Entropy is used to measure the uncertainty of signal distribution and represents the complexity degree of the signal; therefore, information entropy provides a theoretical basis for signal characterization description [18]. In [17], the Renyi Entropy of the Wigner-Ville distribution (WVD) and the continuous wavelet transform (CWT), and the singular spectrum entropy are extracted to identify the 2FSK, BPSK, 16QAM, 32QAM, and MSK signals. The average correct recognition rate of all signals is >90% when SNR> 5 dB. Although the method performs well at low SNRs, it is sensitive to the parameters of the WVD and CWT. Because of underlying periodicities due to various periodic signal processing operations such as sampling, scanning, modulating, multiplexing, and coding, or due to periodicity in the physical phenomenon that gives rise to the time series, many signals can be modeled as cyclostationary signals such as communication signals, radar signals, sonar signals and so on [19,20,21]. The cyclostationary of a signal is usually reflected by the spectral correlation function, also known as the cyclic spectrum. According to [20,21], we can see that the power spectra of different communication signals may be the same, but the cyclic spectra are sometimes significantly different. Moreover, since the noise does not have the characteristics of the cyclostationary, the cyclic spectrum has good anti-noise performance. Based on the advantages mentioned above, the cyclic spectrum is very suitable for identifying communication signals.

In this paper, a novel method for automatic modulation classification of digital communication signals, using SVM based on hybrid features, cyclostationary, and information entropy, is proposed. In this proposed method, by combining the theory of the cyclostationary and entropy, based on the existing signal features, we propose three other new features to assist the classification of communication signals, which are the maximum value of the normalized cyclic spectrum when the cyclic frequency is not zero, the Shannon entropy of the cyclic spectrum, and Renyi entropy of the cyclic spectrum respectively. Since these new features do not require any prior information and have a strong anti-noise ability, they are very suitable for the identification of communication signals. Finally, a one against one SVM is used as a classifier. Simulation results show that the proposed method in this paper performs well in the low SNR condition.

The rest of the paper is arranged as follows: In Section 2, the mathematical model of the communication signals to be identified is given. In Section 3, the proposed features for signal classification in this paper are described in detail. The proposed SVM classifier is given in Section 4. The simulation results are displayed in Section 5. Finally, conclusions are addressed in Section 6.

## 2. Signal Model

In this work, the mathematical model of the signals to be recognized is expressed as

(1) $$\begin{array}{c} y\left(n\right)=x\left(n\right)+v\left(n\right), \end{array}$$

where n=0,1,⋯,N-1, *N* represents the signal length. $y\left(n\right)$, $x\left(n\right)$ and $v\left(n\right)$ are respective the transmitted modulation signal, the intercepted signal, and the noise sample at discrete time *n*. The transmitted signal $\left\{x\left(n\right),n=0,1,\cdots ,N-1\right\}$ is drawn from an unknown constellation set Ψ which in turn belongs to a set of possible modulation formats $\left\{\Psi_{1},\Psi_{2},\cdots ,\Psi_{K}\right\}$. The modulation classification problem refers to the determination of the constellation set Ψ to which the transmitted signal belong based on the intercepted signal $\left\{y\left(n\right),n=0,1,\cdots ,N-1\right\}$. In this paper, we have considered the following digital communication signals for classification: BPSK, EPSK, 2FSK, 4FSK and MSK.

## 3. Feature Extraction

### 3.1. Instantaneous Features

In [3], instantaneous features, which contain hidden modulation information in a single domain, were demonstrated to be suitable for signal classification. According to the considered digital communication signals in this paper, the following instantaneous features are selected.

(1) $\sigma_{ap}$: the standard deviation of the absolute value of the nonlinear component of the instantaneous phase in the non-weak segments of the intercepted signal:
  (2) $$\begin{array}{c} \sigma_{ap}=\sqrt{\frac{1}{C}\left(\sum_{A_{n}\left(n\right)>a_{t}}\phi_{NL}^{2}\left(n\right)\right)-{\left(\frac{1}{C}\sum_{A_{n}\left(n\right)>a_{t}}\left|\phi_{NL}\left(n\right)\right|\right)}^{2}}, \end{array}$$
  where A(n) denotes the instantaneous amplitude and $\phi \left(n\right)$ denotes the instantaneous phase of the intercepted signal both at time instants $t=n/f_{s}$. $A_{n}\left(n\right)=A\left(n\right)/m_{a}$, $m_{a}$ is the average value of the instantaneous amplitude over one frame, that is
  (3) $$\begin{array}{c} m_{a}=\frac{1}{N}\sum_{n=1}^{N}A\left(n\right), \end{array}$$
  $\phi_{NL}\left(n\right)$ is the value of the non-linear component of the instantaneous phase at time instants $t=n/f_{s}$, $a_{t}$ is a threshold for A(n) below which the estimation of the instantaneous phase is very sensitive to the noise, and *C* is the number of samples in $\left\{\phi_{NL}\left(n\right)\right\}$ for which $A_{n}\left(n\right)>a_{t}$. $\sigma_{ap}$ is mainly used to distinguish the MPSK signals and it also can differentiate the modulation schemes of MFSK signals to some extent.
(2) $\sigma_{af}$: standard deviation of the absolute value of the normalized-centered instantaneous frequency over non-weak segments of the intercepted signal:
  (4) $$\begin{array}{c} \sigma_{af}=\sqrt{\frac{1}{C}\left(\sum_{A_{n}\left(n\right)>a_{t}}f_{N}^{2}\left(n\right)\right)-{\left(\frac{1}{C}\sum_{A_{n}\left(n\right)>a_{t}}\left|f_{N}\left(n\right)\right|\right)}^{2}}, \end{array}$$
  where, $f_{N}\left(n\right)=f_{c}\left(n\right)/r_{s}$, $f_{c}\left(n\right)=f\left(n\right)-m_{f}$, $m_{f}=\left(1/N\right)\sum_{i=1}^{N}f\left(i\right)$. $\sigma_{af}$ can differentiate between the modulation types without frequency information and the FSK modulation types and also between 2FSK and 4FSK.

**Figure 1:**
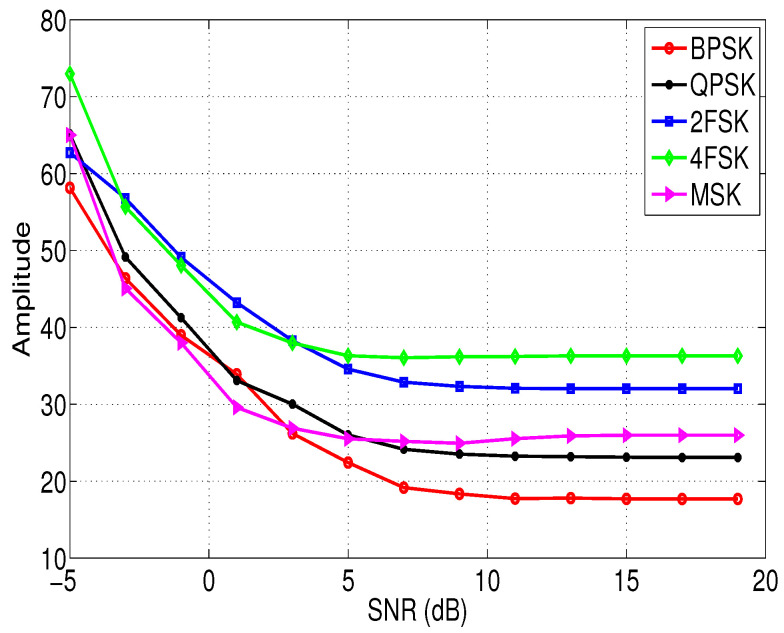
The relationship between the feature $\sigma_{ap}$ of different modulation signals and the SNRs.

**Figure 2:**
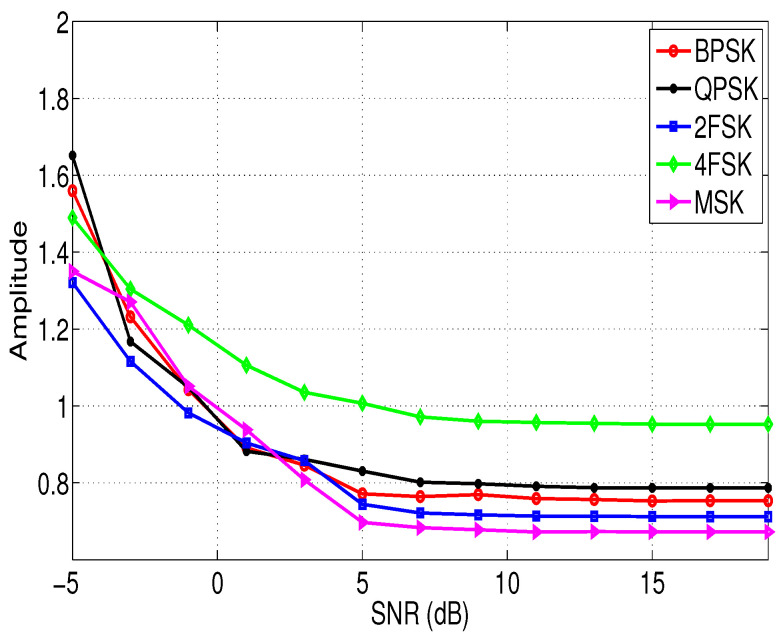
The relationship between the feature $\sigma_{af}$ of different modulation signals and the SNRs.

Figure 1 and Figure 2 show the relationship between the features $\sigma_{ap}$ and $\sigma_{af}$ of different modulation signals with the SNRs. In this simulation, the sampling frequency $f_{s}$ = 10 KHz, the signal length N=4096, and the noise $v\left(n\right)$ is the white Gaussian noise. According to Figure 1 and Figure 2, it is clear that although feature $\sigma_{ap}$ and feature $\sigma_{af}$ of different signals are different, and the difference is not obvious when the SNR is low. Therefore, we need to extract other new features to assist the identification of the modulation types of these signals in low SNR environments, and these features are introduced in the next section.

### 3.2. Features Based on Cyclostationary and Information Entropy

#### 3.2.1. The Cyclostationary of Communication Signals

A discrete time process x(n) is cyclostationary, if the discrete time cyclic autocorrelation function

(5) $$\begin{array}{c} R_{x}^{\alpha }\left(k\right)=\lim_{N\to \infty }\frac{1}{2N+1}\sum_{n=-N}^{N}\left[x\left(n+k\right)e^{-i\pi \alpha (n+k)}\right]{\left[x\left(n\right)e^{i\pi \alpha n}\right]}^{*}, \end{array}$$

exists and is not identically zero when α≠0. A particularly convenient characterization of cyclostationary is the cyclic spectrum,

(6) $$\begin{array}{c} S_{x}^{\alpha }\left(f\right)=\sum_{k=-\infty }^{\infty }R_{x}^{\alpha }\left(k\right)e^{-i2\pi fk}, \end{array}$$

which is the Fourier series transform of the $R_{x}^{\alpha }\left(k\right)$. In (5) and (6), $i=\sqrt{-1}$, * denotes the conjugation operation, α is called the cyclic frequency and *f* is the spectral frequency. Moreover, it is obvious that the cyclic autocorrelation function and the cyclic spectrum will reduce to the conventional autocorrelation function and the power spectral density when the cyclic frequency α=0.

The cyclic spectrum can be calculated directly through the double limit of the time smoothed cyclic periodogram,

(7) $$\begin{array}{c} S_{x}^{\alpha }\left(f\right)=\lim_{T\to \infty }\lim_{\Delta t\to \infty }S_{x_{T}}^{\alpha }{\left(n,f\right)}_{\Delta t}, \end{array}$$

where

(8) $$\begin{array}{c} S_{x_{T}}^{\alpha }{\left(n,f\right)}_{\Delta t}={\langle X_{T}\left(n,f+\alpha /2\right)X_{T}^{*}\left(n,f-\alpha /2\right)\rangle }_{\Delta t}, \end{array}$$

$X_{T}\left(n,f\right)$ is called the complex demodulate of x(n) and is the complex envelop of a narrow-band band-pass component of x(n) centered at *f* with bandwidth Δf≈=1/T,

(9) $$\begin{array}{c} X_{T}\left(n,f\right)=\sum_{m=n-T/2}^{n+T/2}x\left(m\right)e^{-i2\pi fm}, \end{array}$$

where Δf and Δt are called the frequency and time resolutions of the estimation. The time smoothed cyclic periodogram is used to estimate the cyclic spectrum point by point.

When x(n) is the MPSK signal, the mathematical model of x(n) can be described as

(10) $$\begin{array}{c} x\left(n\right)=A\cos\left(2\pi f_{c}n/f_{s}+\phi \left(n\right)\right), \end{array}$$

and

(11) $$\begin{array}{c} \phi \left(n\right)=\sum_{m=-\infty }^{\infty }\theta_{m}q\left(n-mT_{0}\right), \end{array}$$

where $f_{c}$ denotes the carrier frequency, *A* denotes the signal amplitude, $\theta_{m}\in \left\{2k\pi /M,k=0,1,\cdots ,M-1\right\}$ is phase of the transmitted MPSK symbol, $T_{0}$ denotes the symbol period. Here, $q\left(n\right)$ is a rectangle pulse,

(12) $$\begin{array}{c} q\left(n\right)=\left\{\begin{array}{c} 1,\left|n\right|\leq T_{0}f_{s}/2\\ 0,\left|n\right|>T_{0}f_{s}/2 \end{array}\right., \end{array}$$

and therefore

(13) $$\begin{array}{c} Q\left(f\right)=\frac{\sin\left(\pi fT_{0}\right)}{\pi f}. \end{array}$$

When M=2, $x\left(n\right)$ is a BPSK signal, then based on (6) the cyclic spectrum of the BPSK signal can be obtained

(14) $$\begin{array}{c} S_{x}^{\alpha }\left(f\right)=\left\{\begin{array}{c} \frac{1}{4T_{0}}\left[Q\left(f-f_{c}+\frac{\alpha }{2}\right)Q^{*}\left(f-f_{c}-\frac{\alpha }{2}\right)\right.\\ \left.+Q\left(f+f_{c}+\frac{\alpha }{2}\right)Q^{*}\left(f+f_{c}-\frac{\alpha }{2}\right)\right]\\ ,\alpha =p/T_{0}\\ \frac{1}{4T_{0}}Q\left(f-f_{c}+\frac{\alpha }{2}\right)Q^{*}\left(f+f_{c}-\frac{\alpha }{2}\right)\\ ,\alpha =2f_{c}+p/T_{0}\\ \frac{1}{4T_{0}}Q\left(f+f_{c}+\frac{\alpha }{2}\right)Q^{*}\left(f-f_{c}-\frac{\alpha }{2}\right)\\ ,\alpha =-2f_{c}+p/T_{0}\\ 0,else \end{array}\right., \end{array}$$

For all integers *p*. Similarly, when M≥4, the cyclic spectrum of the MPSK signal can be written as

(15) $$\begin{array}{c} S_{x}^{\alpha }\left(f\right)=\left\{\begin{array}{c} \frac{1}{4T_{0}}\left[Q\left(f-f_{c}+\frac{\alpha }{2}\right)Q^{*}\left(f-f_{c}-\frac{\alpha }{2}\right)\right.\\ +\left.Q\left(f+f_{c}+\frac{\alpha }{2}\right)Q^{*}\left(f+f_{c}-\frac{\alpha }{2}\right)\right]\\ ,\alpha =p/T_{0}\\ 0,else \end{array}\right.. \end{array}$$

According to (14) and (15), we can see that the cyclic spectra of the BPSK signals have large values, when $\alpha =p/T_{0}$ and $\alpha =\pm 2f_{c}+p/T_{0}$. However, the cyclic spectra of the MPSK $\left(M\geq 4\right)$ signals only have nonzero values at $\alpha =p/T_{0}$.

Similarly, the type of FSK signals can be expressed as

(16) $$\begin{array}{c} x\left(n\right)=\cos\left[2\pi f_{c}n/f_{s}+\sum_{r=-\infty }^{\infty }\sum_{m=1}^{M}\delta_{m}\left(r\right)\left[2\pi f_{m}\left(n/f_{s}-rT_{0}\right)\right.\left.+\theta_{m}\left(r\right)\right]q\left(n-rT_{0}\right)\right] \end{array},$$

where the *M* frequencies $\left\{f_{c}+f_{m},m=1,2,\cdots ,M\right\}$ are keyed randomly. When the phase sequences $\theta_{m}$ are constant

(17) $$\begin{array}{c} \theta_{r}\left(n\right)=\phi_{m} \end{array},$$

then the FSK is called clock phase coherent FSK and the MSK signal belong to this type of signal. Simultaneously, (16) can be written as

(18) $$\begin{array}{c} x\left(n\right)=\sum_{r=1}^{\infty }\sum_{m=1}^{M}\delta_{m}\left(r\right)q_{m}\left(n-rT_{0}\right) \end{array},$$

where

(19) $$\begin{array}{c} q_{m}\left(n\right)=\cos\left[2\pi \left(f_{c}+f_{m}\right)n/f_{s}+\phi_{m}\right] \end{array},$$

and therefore

(20) $$\begin{array}{c} \begin{array}{c} Q_{m}\left(f\right)=\frac{\sin\left[\pi \left(f-f_{c}-f_{m}\right)T_{0}\right]e^{j\phi_{m}}}{2\pi \left(f-f_{c}-f_{m}\right)}+\frac{\sin\left[\pi \left(f+f_{c}+f_{m}\right)\right]e^{-j\phi_{m}}}{2\pi \left(f+f_{c}+f_{m}\right)} \end{array} \end{array},$$

then based on (6) the cyclic spectrum of (18) can be given by

(21) $$\begin{array}{c} \begin{array}{cc} S_{x}^{\alpha }\left(f\right)= & \frac{1}{MT_{0}}\sum_{m=1}^{M}Q_{m}\left(f+\alpha /2\right)Q_{m}^{*}\left(f-\alpha /2\right)-\\ & \frac{1}{M^{2}T_{0}}\left[\sum_{m=1}^{M}Q_{m}\left(f+\alpha /2\right)\right]{\left[\sum_{n=1}^{M}Q_{n}\left(f-\alpha /2\right)\right]}^{*}\\ & \cdot \left[1-\frac{1}{T_{0}}\sum_{n=-\infty }^{\infty }\delta \left(f+\alpha /2-n/T_{0}\right)\right],\alpha =p/T_{0} \end{array} \end{array},$$

Letting $f_{m}^{{}^{\prime }}=f_{c}+f_{m}$, it can be shown that $\left|S_{x}^{\alpha }\left(f\right)\right|$ has its maximum values at $f=\pm f_{m}^{{}^{\prime }}$, and if $f_{m}^{{}^{\prime }}T_{0}$ are integers, there are additional peaks at $\alpha =\pm f_{m}^{{}^{\prime }}$ and f=0. There are also secondary maxima, down by the factor M-1 from the primary maximum, at $\pm \alpha =f_{m}^{{}^{\prime }}\pm f_{n}^{{}^{\prime }}$ and $\pm f=(f_{m}^{{}^{\prime }}\mp f_{n}^{{}^{\prime }})/2$.

When the phase sequence $\left\{\theta_{m}\left(r\right),m=1,2,\cdots ,M\right\}=\theta_{n}$ is independent and identically distributed with uniform fraction of time distribution over (-π,π], $x\left(n\right)$ in (16) is called phase incoherent FSK and the 2FSK/4FSK signal belong to this type of signal, and (16) can be re-written as

(22) $$\begin{array}{c} x\left(n\right)=\sum_{r=-\infty }^{\infty }\sum_{m=1}^{M}a_{r}\left(n\right)q\left(n-rT_{0}\right) \end{array},$$

and

(23) $$\begin{array}{c} a_{r}\left(n\right)=\cos\left[2\pi \left(f_{c}+f\left(r\right)\right)n/f_{s}+\theta_{r}\right] \end{array},$$

where

(24) $$\begin{array}{c} f\left(r\right)=\sum_{m=1}^{M}\delta_{m}\left(r\right)f_{m} \end{array},$$

For a purely stationary $f\left(r\right)$ with discrete *M*-ary fraction of time distribution ${\left\{P_{m}\right\}}_{1}^{M}$, the cyclic spectrum of (22) can be expressed as

(25) $$\begin{array}{cc} S_{x}^{\alpha }\left(f\right) & =\frac{1}{4T_{0}}\sum_{m=1}^{M}P_{m}\left[Q\left(f+f_{m}^{{}^{\prime }}+\frac{\alpha }{2}\right)Q^{*}\left(f+f_{m}^{{}^{\prime }}-\frac{\alpha }{2}\right)\right.\\ & \left.+Q\left(f-f_{m}^{{}^{\prime }}+\frac{\alpha }{2}\right)Q^{*}\left(f-f_{m}^{{}^{\prime }}-\frac{\alpha }{2}\right)\right],\alpha =p/T_{0} \end{array}.$$

Comparing (21) with (25), we can see that there are no impulses in (18), and there are no peaks at $\alpha =\pm 2f_{m}^{{}^{\prime }}$ for phase incoherent FSK.

**Figure 3:**
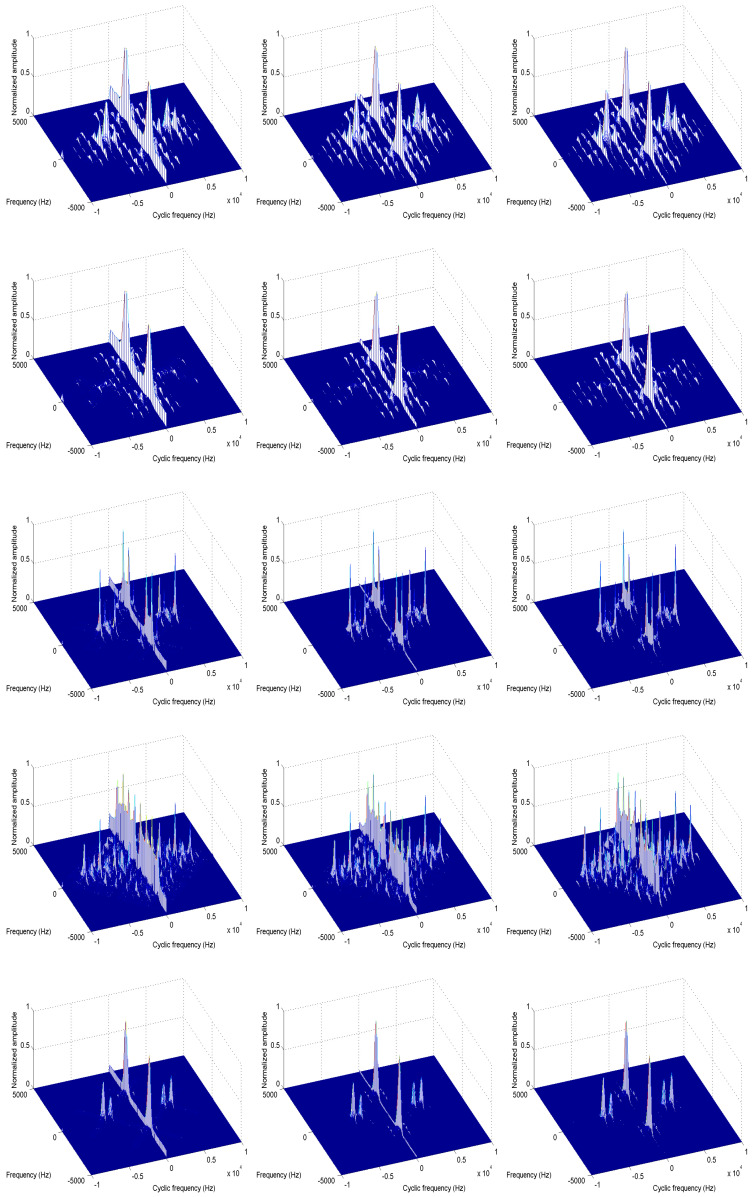
The cyclic spectra of five different modulation signals under different SNRs, the figures from top to bottom show the cyclic spectra of BPSK, QPSK, 2FSK, 4FSK, MSK. The figures in the left, middle and right columns correspond to 0 dB, 5 dB, and 10 dB respectively.

Figure 3 shows the cyclic spectra of different communication signals under different SNR environments. The noise is the additive white Gaussian noise (AWGN). From Figure 3, it is clear that the cyclic spectra (α>0) of these communication signals are not only distinct but also have strong noise immunity, which means the cyclic spectrum is a very good tool for identifying these signals. In this simulation, $f_{s}$ = 10 KHz, N=4096.

#### 3.2.2. The Feature of the Cyclic Spectrum

**Figure 4:**
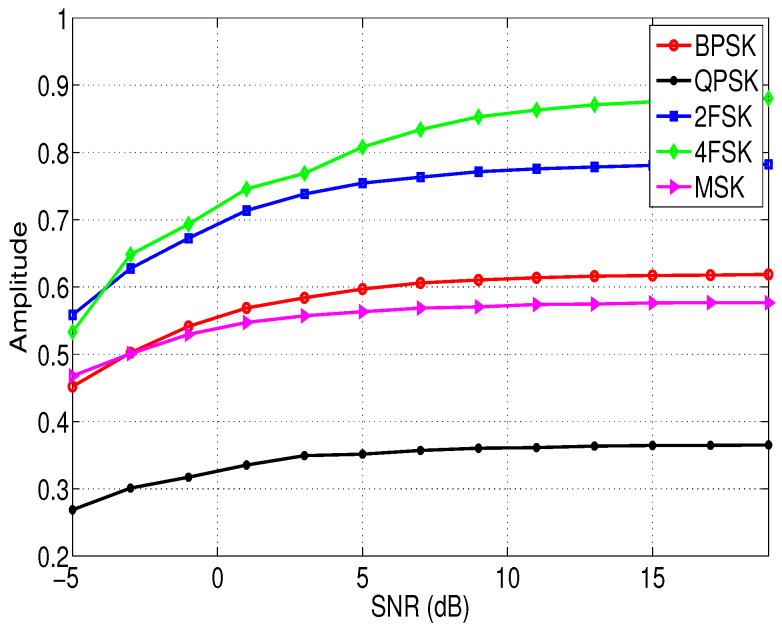
The relationship between the feature $H_{c}$ of different modulation signals and the SNRs.

From Figure 4 and the theory mentioned in Section 3.2.1, we can obtain that the maximum $H_{c}$ of the normalized cyclic spectrum is a good characteristic to distinguish the communication signals, $H_{c}$ is defined as:

(26) $$\begin{array}{c} H_{c}=max\left\{\left|S_{x}^{\alpha >0}\left(f\right)\right|\right\} \end{array}.$$

where max denotes taking the maximum value, || represents taking the amplitude. The relationship between the feature $H_{c}$ of different modulation signals and the SNR is shown in Figure 4. The signal propagation channel is AWGN. It can be seen from Figure 4 that the feature $H_{c}$ of different modulation signals varies significantly, which means $H_{c}$ is a good feature to distinguish them.

#### 3.2.3. The Information Entropy Features of the Cyclic Spectrum

The information entropy was first proposed by Shannon, which was used to measure the uncertainty of signal distribution and represents the complexity degree of the signal. Therefore, information entropy provides a theoretical basis for the signal characterization description. Presently, entropy is applied many subjects [22,23,24,25,26,27,28]. Because of the symmetry of the cyclic spectrum, that is

(27) $$\begin{array}{c} \left|S_{x}^{\alpha }\left(f\right)\right|=\left|S_{x}^{-\alpha }\left(f\right)\right| \end{array}.$$

Then according to [17], the Shannon entropy of the cyclic spectrum can be defined as

(28) $$\begin{array}{c} H_{s}=-\sum_{f}\sum_{\alpha >0}P_{x}^{\alpha }\left(f\right)log_{2}\left(P_{x}^{\alpha }\left(f\right)\right) \end{array},$$

where

(29) $$\begin{array}{c} P_{x}^{\alpha }\left(f\right)=\frac{\left|S_{x}^{\alpha }\left(f\right)\right|}{\sum_{f}\sum_{\alpha }\left|S_{x}^{\alpha }\left(f\right)\right|},\alpha >0 \end{array}.$$

From (28), the entropy $H_{s}$ has several important properties:

(I) Symmetry: when the order of each component $P_{x}^{\alpha }\left(f\right)$ changed, the $H_{s}$ will not be changed, which means the entropy is only related to the whole statistical properties of the data set. According to this property, we can obtain that the entropy $H_{s}$ is robust to the signal modulation parameters such as carrier frequency, code rate, etc.
(II) Non-negative property: the entropy $H_{s}$ is a non-negative value, that is
  (30) $$\begin{array}{c} H_{s}\geq 0 \end{array}.$$
(III) Extreme property: when each component in data set existed in equal probability, the entropy $H_{s}$ will get its maximum value. that is
  (31) $$\begin{array}{c} H_{s}⩽log_{2}\Omega \end{array},$$
  where Ω represents the number of the $P_{x}^{\alpha }\left(f\right)$.

Similarly, according to [17], the two-dimensional Renyi entropy of the cyclic spectrum can be defined as follows:

(32) $$\begin{array}{c} H_{\beta }=\frac{1}{1-\beta }log_{2}\sum_{f}\sum_{\alpha >0}{\left(P_{x}^{\alpha }\left(f\right)\right)}^{\beta } \end{array},$$

where β is the order of the Renyi entropy of the cyclic spectrum, and β≥0,β≠1. Compared with Shannon entropy, the Renyi entropy can better reflect the difference between two different distributions [29].

**Figure 5:**
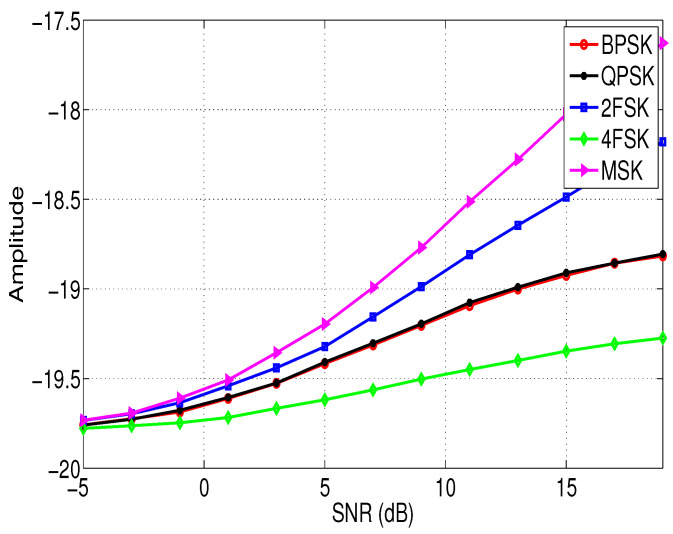
The relationship between the entropy feature $H_{s}$ of different modulation signals and the SNRs.

**Figure 6:**
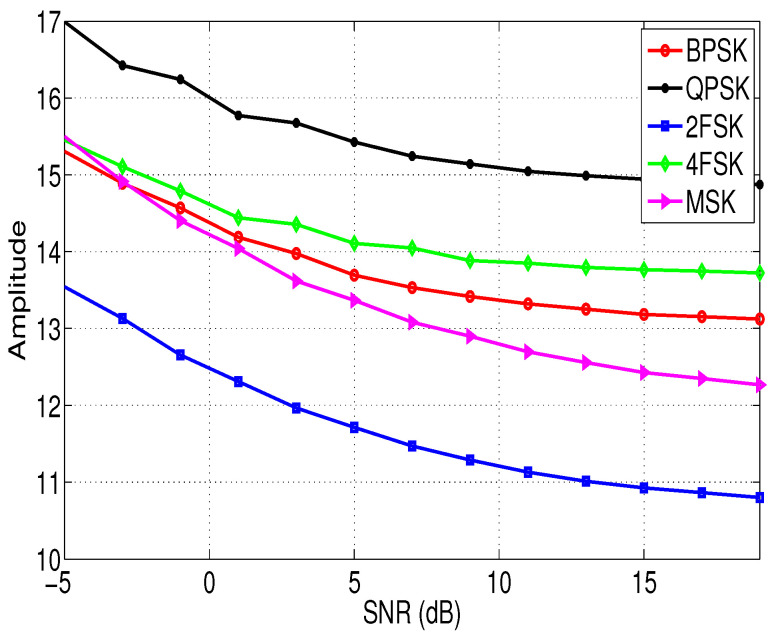
The relationship between the entropy feature $H_{\beta }$ of different modulation signals and the SNRs.

The relationship between the information entropy $H_{s}$ and $H_{\beta }$ of different modulation signals and the SNR is shown in Figure 5 and Figure 6. In these simulations, the sampling frequency $f_{s}$ = 10 KHz, the signal length N=4096, and without loss of generality, the order β is set to 5. The noise $v\left(n\right)$ is the white Gaussian noise. From Figure 5, it is clear that the entropy $H_{s}$ is a good feature to distinguish 2FSK, 4FSK, and MSK in a low SNR environment. Similarly, the entropy $H_{\beta }$ is a good feature to distinguish between BPSK and QPSK, 4FSK and 2FSK, 4FSK and MSK when the SNR is low.

## 4. The Proposed SVM Classifier

The traditional artificial neural networks (ANNs) often encounter problems such as overfitting and local minimization. Meanwhile, the large amount of sample data needed for full training of an ANN cannot be guaranteed in practical applications [30,31,32]. The SVM based on the structural risk minimization criterion cannot only minimize the classification error but also improve the generalization ability and has outstanding small sample learning ability. Therefore, based on the mentioned above, this paper will use the SVM to design the classifier to automatically identify the types of the modulation signals.

Given a training set of instance-label pairs $\left(x_{i},y_{i}\right),i=1,2,\cdots ,l$ where $x_{i}\in R^{n}$ is the input vector and $y_{i}\in {\left\{1,-1\right\}}^{l}$ represents two classes label. Then the mathematical model for the two classes of SVM classifiers can be defined as follows:

(33) $$\begin{array}{c} \min_{w,b,\xi }\frac{1}{2}{∥w∥}^{2}+D\sum_{i=1}^{l}\xi_{i}\\ s.t\;y_{i}\left(w^{T}\Phi \left(x_{i}\right)+b\right)⩾1-\xi_{i} \end{array},$$

where i=1,2,⋯,l, *w* is the vector of weight coefficient, $\xi_{i}⩾0$ is the slack variable for the errors, D>0 is the penalty parameter of the error term, a larger *D* corresponding to assigning a higher penalty to errors.

Each $x_{i}$ is then mapped to a $\Phi \left(x_{i}\right)$ in the kernel-induced feature space, which is related to the kernal function

(34) $$\begin{array}{c} K\left(x_{i},x_{j}\right)=\Phi {\left(x_{i}\right)}^{T}\Phi \left(x_{j}\right) \end{array}.$$

Then the standard SVM tries to find a hyperplane $w^{T}\Phi \left(x\right)+b$ that has a large margin and small training error. The kernel function has many types, such as linear function, polynomial function, radial basis function (RBF), and sigmoid function. The expressions of these functions are given as follows [33]:

(I) The linear kernel function:
  (35) $$\begin{array}{c} K\left(x_{i},x_{j}\right)=\gamma x_{i}^{T}x_{j} \end{array}.$$
(II) The polynomial kernel function:
  (36) $$\begin{array}{c} K\left(x_{i},x_{j}\right)={\left(\gamma x_{i}^{T}x_{j}+r\right)}^{d} \end{array}.$$
(III) The RBF kernel function:
  (37) $$\begin{array}{c} K\left(x_{i},x_{j}\right)=e^{-\gamma {∥x_{i}-x_{j}∥}^{2}} \end{array}.$$
(IV) The sigmoid kernel function:
  (38) $$\begin{array}{c} K\left(x_{i},x_{j}\right)=tanh\left(\gamma x_{i}^{T}x_{j}+r\right) \end{array}.$$
  where γ is the reciprocal of the number of signal types to be classified. Obviously, in this paper γ=1/5. The effect of these kernel functions on the classification performance of SVM is discussed in detail in the next section.

**Figure 7:**
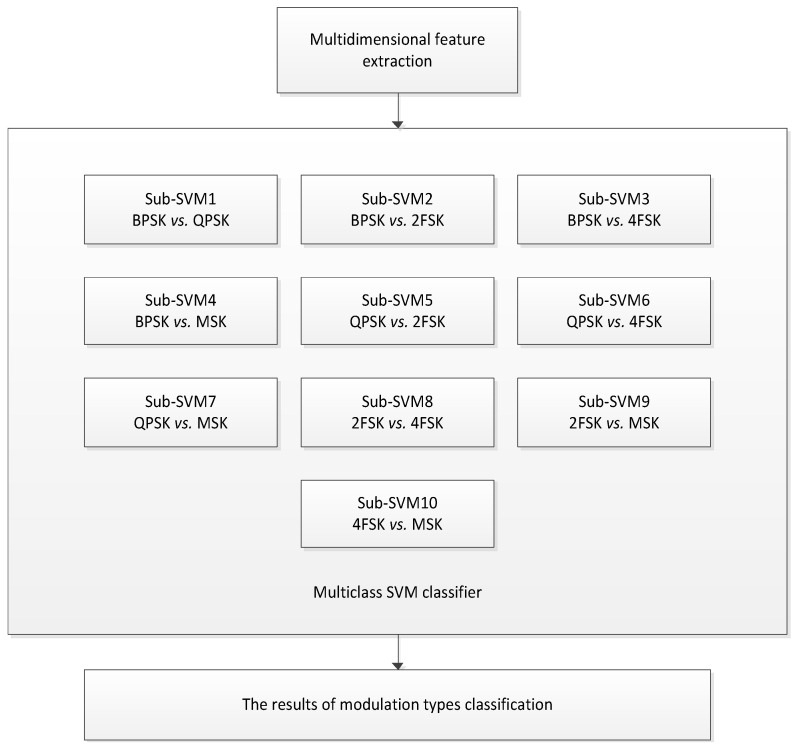
The structure of multiclass SVM classifier based on ten sub-SVMs using the one-versus-one algorithm.

SVM was originally only used for two types of classification problems, in order to achieve multi-classification problems a multiclass SVM comprising ten two-class sub-SVMs is designed. The number of sub-SVMs is U(U-1)/2, where *U* is the number of the signal types. Figure 7 shows the classification procedure structure of the multiclass SVM proposed in this paper.

## 5. Simulation Results

This section shows the simulation results of the proposed method for the classification of the considered digital modulation signals {BPSK,QPSK,2FSK,4FSK,MSK}, and the feature set adopted in these tests is $\left\{\sigma_{ap},\sigma_{af},H_{c},H_{s},H_{\beta }\right\}$. The sampling frequency $f_{s}$ = 10 KHz, and the signal length *N* = 4096. The noise is the additive white Gaussian noise and was added according to SNRs {−5 dB,−4 dB, ⋯, 20 dB}. Each modulation type has 2000 realizations and half of the realizations with SNRs of −5 dB, 0 dB, 5 dB, 15 dB, and 20 dB are used for training. Simulations results have been given in figures and tables, and we use the accuracy metric to test the recognition performance. Furthermore, we have given some confusion matrixes for particular experiments that are considerable.

### 5.1. Classification in AWGN Channel

Table 1, Table 2 and Table 3 show the confusion matrixes over the AWGN channel of the proposed methods under different SNRs. The kernel function used here is the RBF function. From these tables, we can obtain that the overall accuracy of the proposed method for different modulation signals can reach 85.92%, when the SNR = 0 dB, and the overall accuracy will be greater than 99% when the SNR≥ 6 dB.

To evaluate the performance of different kernel functions for multiclass SVM. Table 4 shows the overall accuracy of the proposed method when using different kernel functions, and the SNR = 6 dB. According to Table 4, it is obvious that for the method proposed in this paper, the RBF function has the best performance, and the Sigmoid function has the worst performance. Therefore, the RBF function is recommended for the kernel function of the SVM classifier designed in this paper.

In practical applications, the complexity of the algorithm is an important consideration. Then, in order to measure the computational complexity of the proposed method in this paper, the recognition time of each sub-SVM is shown in Table 5. The simulation is implemented on a computer with a CPU of Intel Core 2.6 GHz i5-3230M and 4-Gb RAM, under the 64-bit Windows 7 system (Microsoft, Redmond, WA, USA). The multiclass SVM is accomplished via MATLAB2011b (MathWorks, Natick, MA, USA). In practice, it is easy to find DSP with similar performance, such as TMS320C6678 and so on. Since the proposed SVM classifier in this paper uses a parallel structure, the time spent by the SVM classifier is equal to the maximum time spent by one of the sub-SVMs. From Table 5, we can obtain that the maximum time of the sub-SVMs is 35.708 μs, which is acceptable in practical applications.

**Figure 8:**
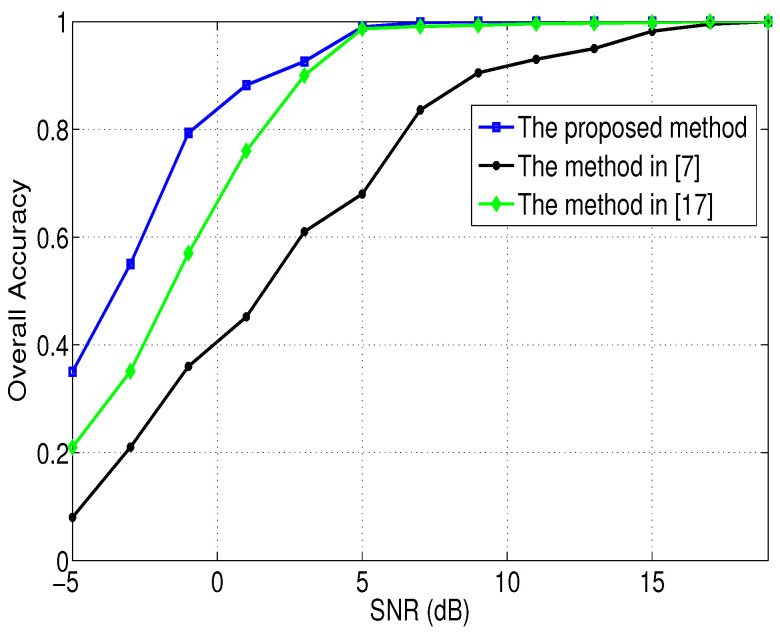
The overall accuracy of different methods under different SNRs.

To show the superiority of the method proposed in this paper, the performance of the proposed method is investigated by making comparisons with the existing methods in [7,17]. Figure 8 shows the overall accuracy of different methods under different SNRs. The test uses 1000 Monte Carlo experiments. According to Figure 8, we can obtain that when the SNR< 5 dB, the recognition performance of the method proposed in this paper is better than that of the methods in [7,17], and when the SNR≥ 5 dB, the recognition performance of the method proposed in this paper is comparable to that of the method in [17].

### 5.2. Classification in Fading Channel

In practical environments, the propagation of signals is often affected by the channels. Table 6 shows the performance of the proposed method when the channel is the Rayleigh channel, and the SNR = 6 dB. It is assumed that there are two channels of multipath signals, the delay of multipath signals is 0.005 s and 0.01 s respectively, and the frequency deviation of multipath signals is 5 Hz and 10 Hz respectively. From Table 6 we can see that the overall accuracy is 99.22% on this occasion, which is comparable to that shown in Table 2. This is because since the multipath effect will affect the amplitude of the cyclic spectrum but not the shape of the cyclic spectrum, the multipath effect has little influence on the entropy characteristics proposed in this paper, so at this point, the performance of the method presented in this paper will not be seriously affected.

## 6. Conclusions

Since digital communication signals are widely used in various military and civilian applications, it is important to improve the recognition rate of digital communication signals. In this paper, a novel signal classification method using SVM based on hybrid features, cyclostationary, and information entropy is proposed. The method combines the theory of the cyclostationary and entropy and uses a one against one SVM as a classifier. Simulation results show that the proposed method has a good recognition performance for the signals considered in this paper when in low SNR environments and fading channels.

## Figures and Tables

**Table 1:** The confusion matrix over AWGN channel, SNR = 0 dB.

Actual Modulation Type	Predicted Modulation Type				
	BPSK	QPSK	2FSK	4FSK	MSK
BPSK	912	0	0	4	128
QPSK	0	954	0	0	0
2FSK	2	0	856	130	76
4FSK	0	0	4	786	8
MSK	86	46	140	80	788
Overall Accuracy	85.92%				

**Table 2:** The confusion matrix over AWGN channel, SNR = 6 dB.

Actual Modulation Type	Predicted Modulation Type				
	BPSK	QPSK	2FSK	4FSK	MSK
BPSK	1000	0	0	0	15
QPSK	0	998	0	0	0
2FSK	0	2	1000	4	7
4FSK	0	0	0	996	0
MSK	0	0	0	0	978
Overall Accuracy	99.44%				

**Table 3:** The confusion matrix over AWGN channel, SNR = 12 dB.

Actual Modulation Type	Predicted Modulation Type				
	BPSK	QPSK	2FSK	4FSK	MSK
BPSK	1000	0	0	0	3
QPSK	0	1000	0	0	0
2FSK	0	0	1000	2	0
4FSK	0	0	0	998	0
MSK	0	0	0	0	997
Overall Accuracy	99.9%				

**Table 4:** Comparison of different kernel functions for overall accuracy over AWGN channel, SNR = 6 dB.

Kernel Function	Overall Accuracy
Linear function	98.65%
Polynomial function	99.32%
RBF	99.44%
Sigmoid	89.6%

**Table 5:** Each sub-SVM recognition time in the real-time recognition system.

Sub-SVM	Time (μs)
BPSK vs. QPSK	9.4673
BPSK vs. 2FSK	1.4247
BPSK vs. 4FSK	4.9047
BPSK vs. MSK	35.708
QPSK vs. 2FSK	3.05
QPSK vs. 4FSK	1.0533
QPSK vs. MSK	1.6783
2FSK vs. 4FSK	2.2073
2FSK vs. MSK	2.494
4FSK vs. MSK	1.0521
Maximum time	35.708

**Table 6:** The confusion matrix over Rayleigh channel, SNR = 6 dB.

Actual Modulation Type	Predicted Modulation Type				
	BPSK	QPSK	2FSK	4FSK	MSK
BPSK	975	6	0	0	4
QPSK	17	994	0	0	0
2FSK	0	0	999	2	1
4FSK	0	0	0	998	0
MSK	8	0	1	0	995
Overall Accuracy	99.22%				
